# Supplementary material for: Total RNA sequencing reveals gene expression and microbial alterations shared by oral pre-malignant lesions and cancer
Source: bioRxiv. 2023 Mar 24:2023.03.24.534064. Preprint. [Version 1] doi: 10.1101/2023.03.24.534064 (PMC10055367; doi:10.1101/2023.03.24.534064)
Supplement: Supplement 1 [file NIHPP2023.03.24.534064v1-supplement-1.pdf]

bioRxiv preprint doi: https://doi.org/10.1101/2023.03.24.534064; this version posted March 24, 2023. The copyright holder for this preprint (which was not certified by peer review) is the author/funder, who has granted bioRxiv a license to display the preprint in perpetuity. It is made available under aCC-BY 4.0 International license.

SUPPLEMENTARY FIGURES:

Supplementary Figure 1:

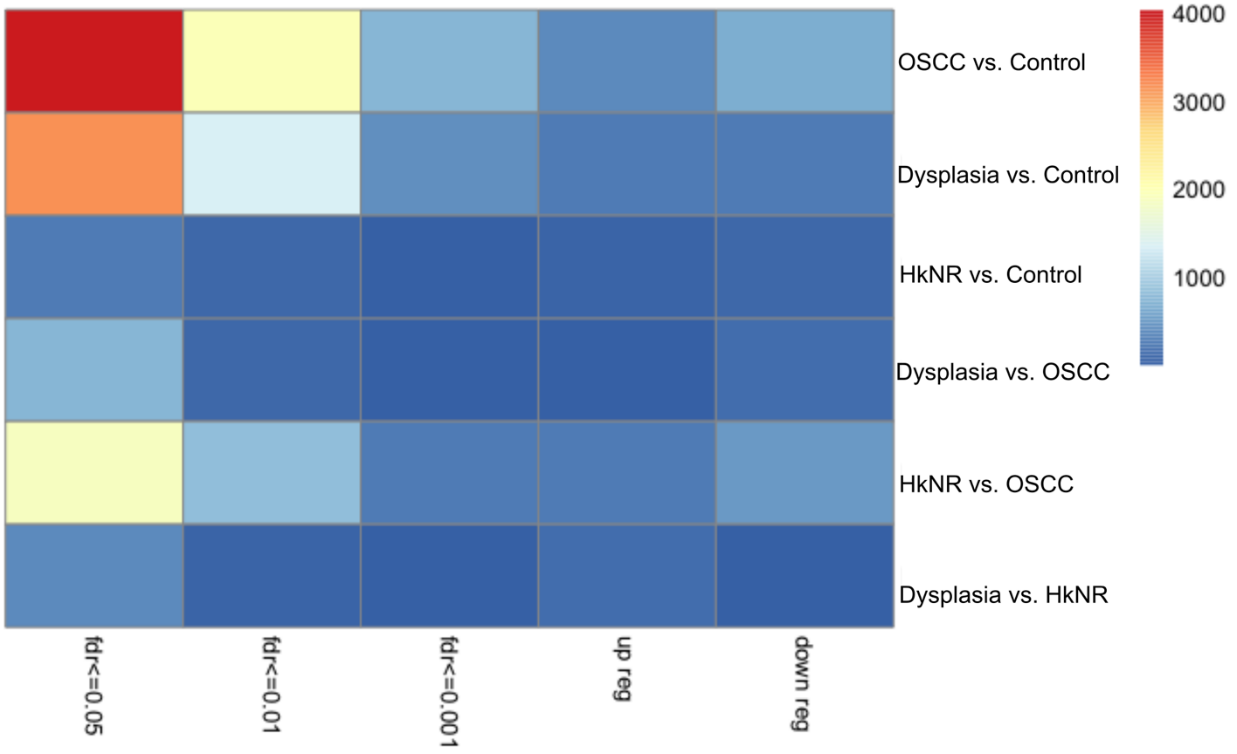

bioRxiv preprint doi: https://doi.org/10.1101/2023.03.24.534064; this version posted March 24, 2023. The copyright holder for this preprint (which was not certified by peer review) is the author/funder, who has granted bioRxiv a license to display the preprint in perpetuity. It is made available under aCC-BY 4.0 International license.

Supplementary Figure 2:

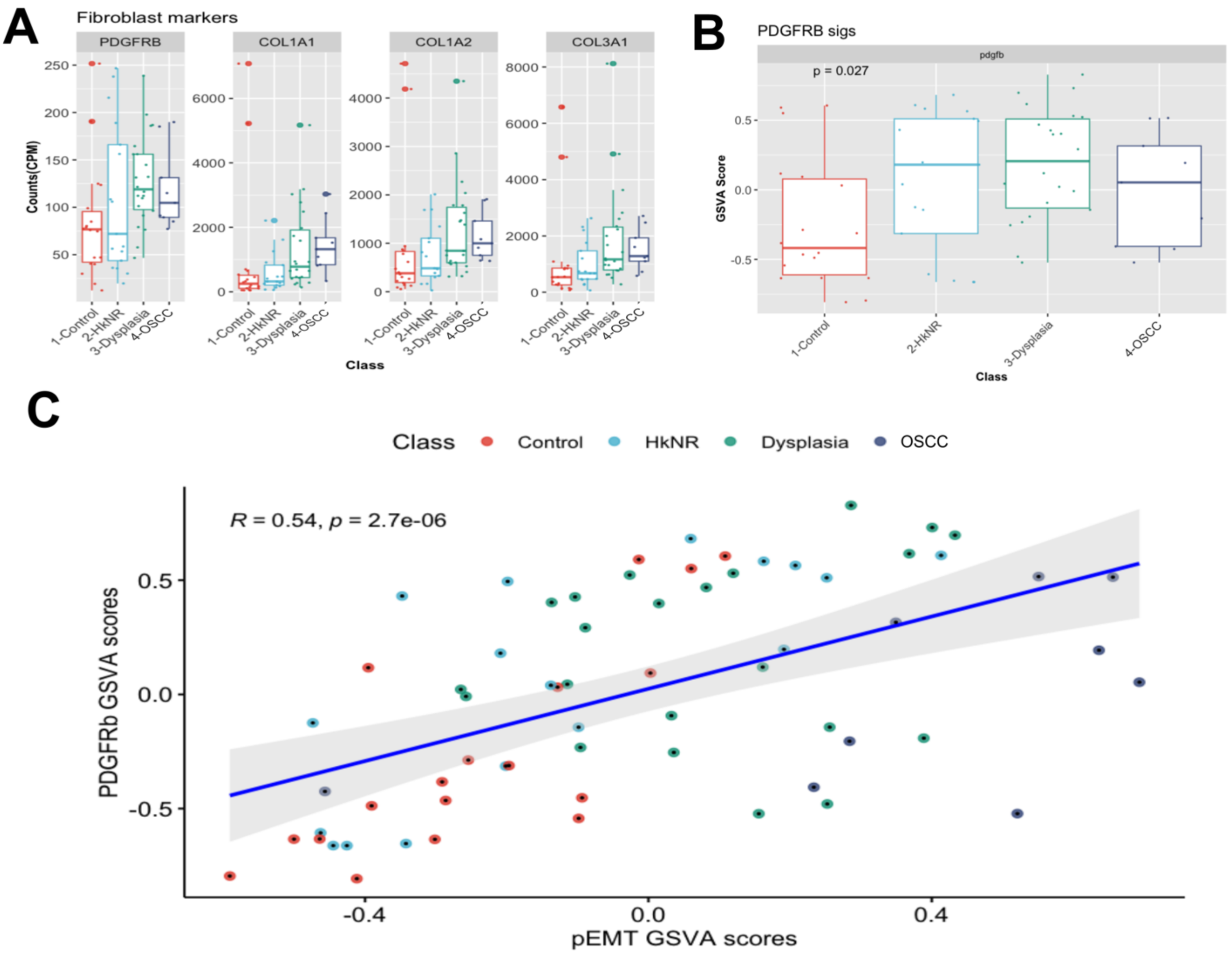

Supplementary Figure 3:

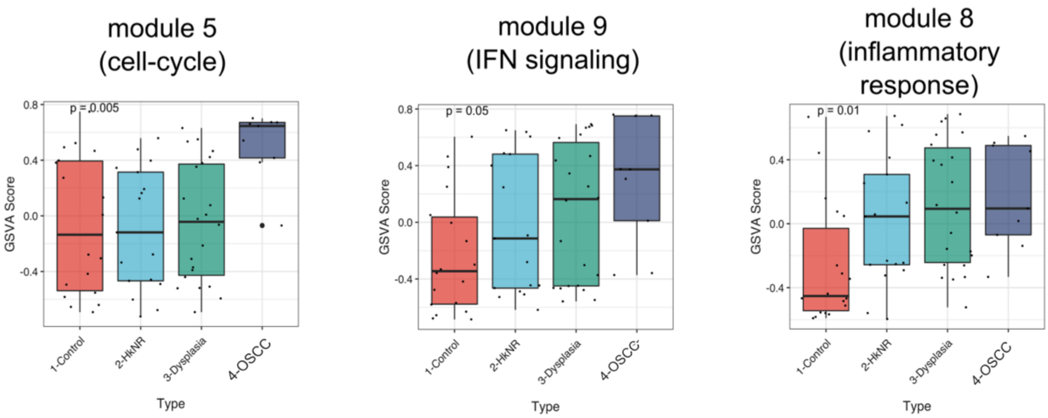

bioRxiv preprint doi: https://doi.org/10.1101/2023.03.24.534064; this version posted March 24, 2023. The copyright holder for this preprint (which was not certified by peer review) is the author/funder, who has granted bioRxiv a license to display the preprint in perpetuity. It is made available under aCC-BY 4.0 International license.

Supplementary Figure 4:

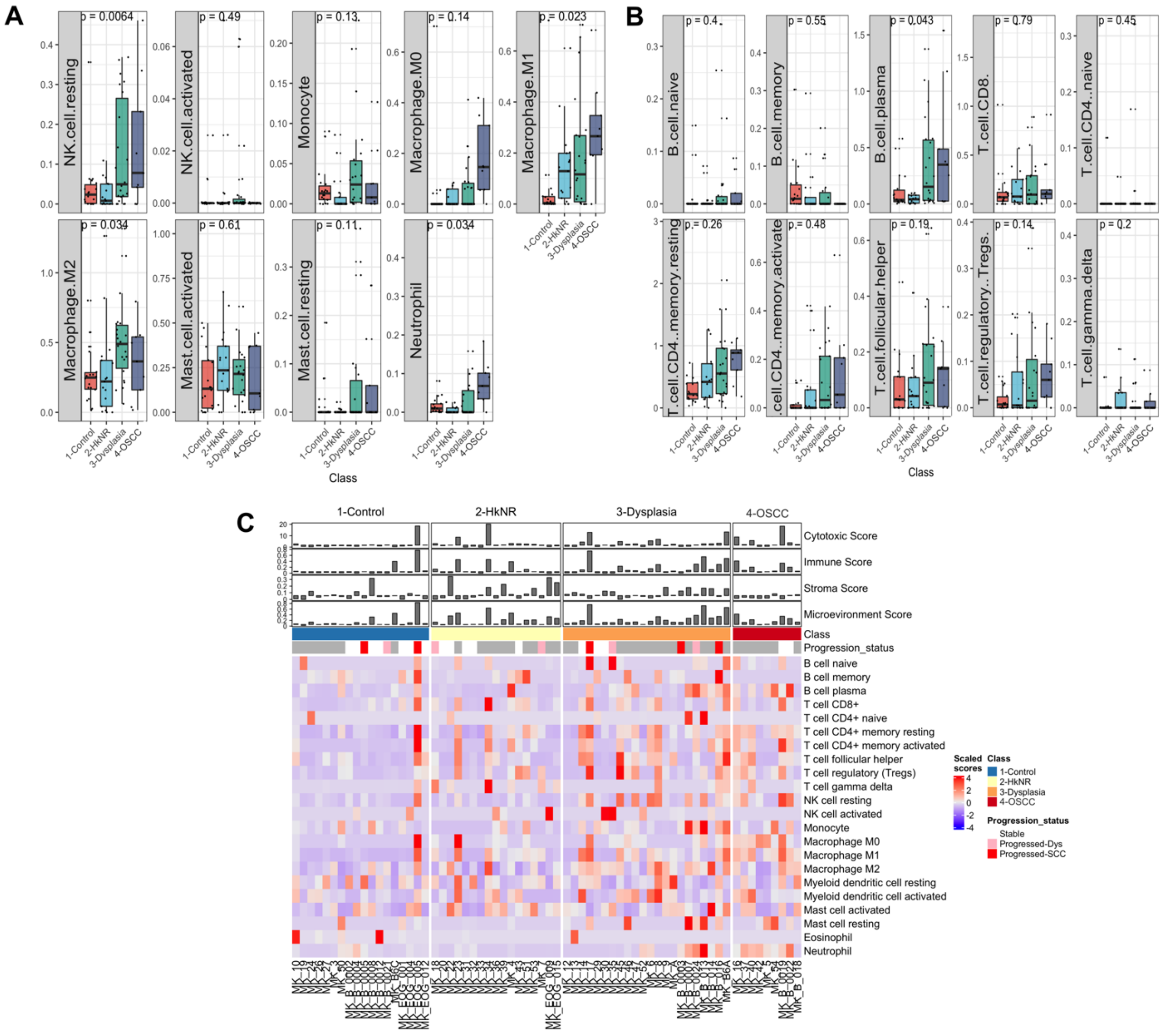

bioRxiv preprint doi: https://doi.org/10.1101/2023.03.24.534064; this version posted March 24, 2023. The copyright holder for this preprint (which was not certified by peer review) is the author/funder, who has granted bioRxiv a license to display the preprint in perpetuity. It is made available under aCC-BY 4.0 International license.

Supplementary Figure 5:

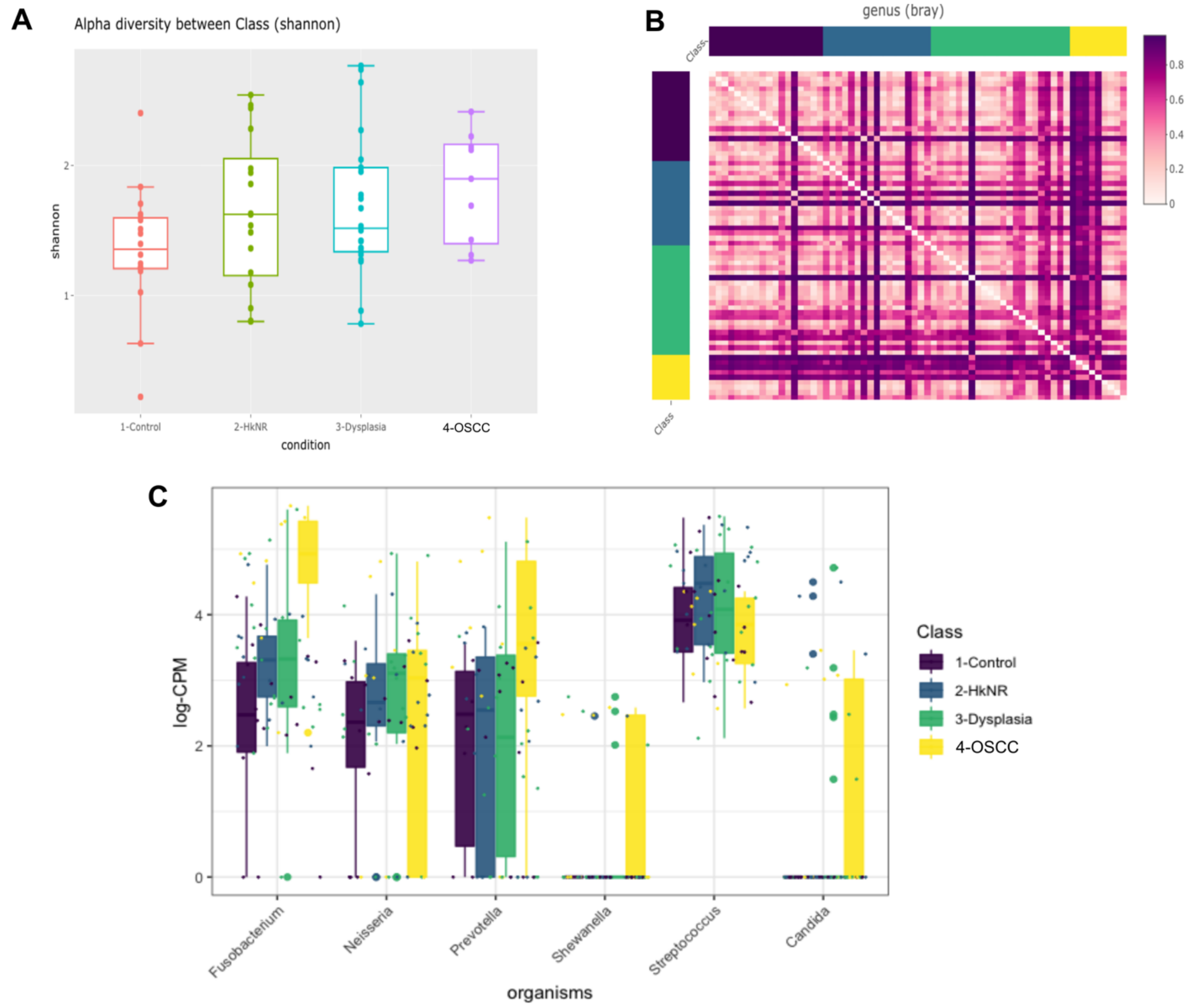

Supplementary Figures

S1. Total number of markers per pairwise analysis. Total number of differentially expressed genes in the pairwise analysis with logFC $\pm$ 1.5 and q-val $\leq$ {0.01, 0.05, 0.1}

S2. Enrichment of cancer-associated fibroblasts. A. Gene expression of PDGFB1, COL1A1, COL1A2, COL3A1. B. Top 50 genes from fibroblast signatures shows enrichment in PML and OSCC groups. C. Association of p-EMT and fibroblasts GSVA scores.

bioRxiv preprint doi: https://doi.org/10.1101/2023.03.24.534064; this version posted March 24, 2023. The copyright holder for this preprint (which was not certified by peer review) is the author/funder, who has granted bioRxiv a license to display the preprint in perpetuity. It is made available under aCC-BY 4.0 International license.

S3. Enrichment of lung bronchus modules. GSVA enrichment scores from modules 5, 8, and 9 from lung bronchus PML study.

S4. Cell-type deconvolution scores of immune sub-types A. innate, B. adaptive types. C. heatmap of abundances stratified by histopathology along with smoking and progression statuses.

S5. Microbial diversity analysis. A. Alpha diversity stratified by histopathological groups. B. Beta diversity stratified by histopathological groups. C. relative abundance in logCPM counts for selected disease-causing genera.

Supplementary Tables

ST1. Differential expression analysis results from DESeq2 for pairwise comparisons between the histopathological groups.

ST2. Pathway enrichment analysis of differentially expressed genes pairwise using hyper enrichment analysis using Hallmark compendium.

ST3. Pathway enrichment analysis of differentially expressed genes pairwise using hyper enrichment analysis using Reactome compendium.

ST4. Microbial differential abundant analysis results from DESeq2 for pairwise comparisons between the histopathological groups.

ST5. MSEA results of microbe-set associations with genes.

ST6. Hyper enrichment analysis of microbe-set genes from MSEA on enriched pathways from host analysis.
